# Assessment of the Humoral Immune Response Following COVID-19 Vaccination in Healthcare Workers: A One Year Longitudinal Study

**DOI:** 10.3390/biomedicines10071526

**Published:** 2022-06-28

**Authors:** Mihaela Chivu-Economescu, Teodora Vremera, Simona Maria Ruta, Camelia Grancea, Mihaela Leustean, Daniela Chiriac, Adina David, Lilia Matei, Carmen C. Diaconu, Adina Gatea, Ciprian Ilie, Iuliana Radu, Ana Maria Cornienco, Luminita Smaranda Iancu, Catalin Cirstoiu, Corina Silvia Pop, Radu Petru, Victor Strambu, Stefan Malciolu, Corneliu Petru Popescu, Simin Aysel Florescu, Alexandru Rafila, Florentina Ligia Furtunescu, Adriana Pistol

**Affiliations:** 1 Stefan S. Nicolau Institute of Virology, Romanian Academy, 030304 Bucharest, Romania; mihaela.chivu@gmail.com (M.C.-E.); cgrancea@yahoo.co.uk (C.G.); dana.chiriac1976@gmail.com (D.C.); liliamatei@yahoo.com (L.M.); ccdiaconu@yahoo.com (C.C.D.); 2 National Institute of Public Health Bucharest, 050463 Bucharest, Romania; teodora.vremera@insp.gov.ro (T.V.); mihaela.leustean@insp.gov.ro (M.L.); adina.david@insp.gov.ro (A.D.); adina.gatea@insp.gov.ro (A.G.); ciprian.ilie@insp.gov.ro (C.I.); iuliana.radu@insp.gov.ro (I.R.); cornienco.anamaria@insp.gov.ro (A.M.C.); 3 ECDC Fellowship Programme, Public Health Microbiology Path (EUPHEM), European Centre for Disease Prevention and Control (ECDC), 16973 Solna, Sweden; 4 Faculty of Medicine, Carol Davila University of Medicine and Pharmacy, 050474 Bucharest, Romania; catalin.cirstoiu@umfcd.ro (C.C.); cora.pop@umfcd.ro (C.S.P.); drradupetru@yahoo.com (R.P.); victor.strambu@umfcd.ro (V.S.); corneliu.popescu@umfcd.ro (C.P.P.); simin.florescu@umfcd.ro (S.A.F.); alexandru.rafila@umfcd.ro (A.R.); florentina.furtunescu@umfcd.ro (F.L.F.); adriana.pistol@umfcd.ro (A.P.); 5 Faculty of Medicine, “Grigore T. Popa” University of Medicine and Pharmacy, 700115 Iasi, Romania; luminita.iancu@umfiasi.ro; 6 Regional Center of Public Health Iași, 700465 Iași, Romania; 7 University Emergency Hospital, 050098 Bucharest, Romania; 8 Dr. Carol Davila Nephrology Clinical Hospital, 010731 Bucharest, Romania; 9 Victor Babes Hospital for Infectious and Tropical Diseases, 030303 Bucharest, Romania; smalciolu@gmail.com; 10 National Institute of Infectious Diseases “Matei Bals”, 021105 Bucharest, Romania

**Keywords:** SARS-CoV-2, vaccination, booster dose, healthcare workers, immune response, host factors, breakthrough infections

## Abstract

The continuous variability of SARS-CoV-2 and the rapid waning of specific antibodies threatens the efficacy of COVID-19 vaccines. We aimed to evaluate antibody kinetics one year after SARS-CoV-2 vaccination with an mRNA vaccine in healthcare workers (HCW), with or without a booster. A marked decline in anti-Spike(S)/Receptor Binding Domain (RBD) antibody levels was registered during the first eight months post-vaccination, followed by a transitory increase after the booster. At three months post-booster an increased antibody level was maintained only in HCW vaccinated after a prior infection, who also developed a higher and long-lasting level of anti-S IgA antibodies. Still, IgG anti-nucleocapsid (NCP) fades five months post-SARS-CoV-2 infection. Despite the decline in antibodies one-year post-vaccination, 68.2% of HCW preserved the neutralization capacity against the ancestral variant, with a decrease of only 17.08% in the neutralizing capacity against the Omicron variant. Nevertheless, breakthrough infections were present in 6.65% of all participants, without any correlation with the previous level of anti-S/RBD IgG. Protection against the ancestral and Omicron variants is maintained at least three months after a booster in HCW, possibly reflecting a continuous antigenic stimulation in the professional setting.

## 1. Introduction

The newly emerged Severe Acute Respiratory Syndrome Coronavirus 2 (SARS-CoV-2) caused an unprecedented global pandemic with more than 513 million confirmed cases and 6.2 million deaths by 1 May 2022. In Romania, as of 1 May 2022, there are more than 2.8 million confirmed cases and 65,000 deaths [1,2].

Most cases of COVID-19 are mild to moderate, and those infected recover without specific treatment; however, severe disease can occur in various proportions, especially in the elderly and those with underlying medical conditions [3,4]. In addition, convalescent patients, irrespective of the clinical form of the acute infection, can experience persistent, variable symptomatology generally defined as long COVID-19, including newly diagnosed cardiovascular, metabolic, autoimmune, or neurologic diseases. As such, prevention measures are needed to reduce viral exposure. Healthcare workers (HCW) represent a particularly vulnerable group due to their essential role in patients’ care and their increased and continuous exposure to infection. Vaccination is a critical prophylaxis measure, aimed to ensure not only the safety of HCW and their patients, but also to interrupt the chains of viral transmission in the community [5]. As such, HCW were prioritized by most countries for vaccination at the beginning of 2021. The currently approved vaccines are highly efficient in protection against hospitalization and severe infection; nevertheless, their efficacy seems to diminish over time, especially against symptomatic infection and breakthrough infections in vaccinated people, including HCW.

The humoral immune response to COVID-19 vaccines is influenced by a large number of factors: previous COVID-19 infection, age, immunosuppression, type of vaccine, the number of doses received, and time since vaccination [5,6,7]. Longer intervals between the first and second dose of an mRNA vaccine correlate with stronger and more persistent immune responses [8]. Information regarding the duration of immunity after vaccination is not yet conclusive; it remains to be determined if a decrease in serum antibody levels can be used to indicate the degree of protection and the necessity of booster shots. It is hypothesized that protection is less influenced by waning antibody levels and instead is more dependent on the initial immune response and the emergence of viral variants able to evade pre-existing immunity [9]. In previous studies of the dynamics of immune response in HCW six months after vaccination and/or infection, we and other research groups reported that the best protection is conferred by vaccination in those with prior infection, via the development of persistent and more broadly neutralizing antibodies as well as stronger cellular immune responses compared to those elicited by a two-dose vaccination regimen only [10,11,12,13].

Medium- and long-term follow-ups of the kinetics of the immune response after COVID-19 vaccination are necessary to predict the level of protection and adjust protective measures for different risk groups [5,6,7].

**AIM:** To evaluate the dynamics and persistence of the humoral immune response in healthcare workers one year after SARS-CoV-2 vaccination with an mRNA vaccine, and to examine the contribution of host factors that may influence the humoral immune response (gender, age, comorbidities, BMI, and previous SARS-CoV-2 infection).

## 2. Materials and Methods

### 2.1. Participant Selection and Data Collection

HCW from four hospitals in Bucharest, Romania (Victor Babes Hospital for Infectious and Tropical Diseases, University Emergency Hospital, Dr. Carol Davila Nephrology Clinical Hospital, and Stefan S. Nicolau Institute of Virology), volunteered to participate in this 12 month prospective longitudinal observational study to determine the dynamics of antibody levels after anti-COVID-19 vaccination. At the time of enrollment, all participants were vaccinated with two doses of the BNT162b2 mRNA vaccine (Pfizer-BioNTech).

Convenience sampling was used to select participants from designated hospitals. People were voluntarily enrolled in the study after receiving a complete package of information about the study and signing the informed consent. The protocol was approved by the Ethics Commission of the National Institute of Public Health (No 3245/05.03.2021). The group was followed up between March 2021 and January 2022.

The sample size was calculated using Epi Info software, taking into consideration the number of healthcare workers vaccinated (537 healthcare workers), with a confidence level of 95% and considering a maximum loss to follow-up of 40%. A total of 945 individuals were initially enrolled in the study, of whom 571 had at least two adequate blood samples collected for analysis at proper intervals. Information regarding age, sex, weight, height, and comorbidities was collected from questionnaires completed by the participants, along with data regarding the existence of a prior SARS-CoV-2 infection, the date of diagnosis, and the form of the disease.

Venous blood samples were collected at 1 month, 3 months, 6 months, 9 months, and 12 months after the second dose. Prior to the 9-month collection, a booster was recommended at the national level, and approximately 70% of study participants received a third dose of BNT162b2 mRNA vaccine (Pfizer-BioNTech).

### 2.2. Antibody Levels Measurement

Detection of anti-S/RBD IgG was accomplished using a chemiluminescent microparticle immunoassay (SARS-CoV-2 IgG II Quant Abbott, ARCHITECT i System, Chicago, IL, USA), with 99.35% sensitivity and 99.60% specificity, according to the manufacturer. Antibody levels were measured in arbitrary units (AU/mL) and the results were converted to binding antibody units (1 BAU/mL = 0.142 × AU/mL) using the manufacturer’s conversion factor for the WHO International Standard. A result was considered positive at values greater than 50 AU/mL (7 BAU/mL).

IgG antibodies against the nucleocapsid protein (NCP) were tested using a qualitative chemiluminescent test (Abbott SARS-CoV-2 IgG assay on ARCHITECT i System) with sensitivity >99.5% and specificity >99.5%. A positive result was considered at an index ratio ≥ 1.4.

IgA antibodies against Spike protein were determined using an enzyme-linked immunosorbent assay (EUROIMMUN Medizinische Labordiagnostika AG, Lübeck, Germany) with a sensitivity >90% and specificity >98.3%. A positive result was considered at an index ratio ≥ 1.1, calculated as the OD 450 of the subject sample over the OD 450 of a calibrator (an anti-S human IgA provided by the manufacturer).

### 2.3. Surrogate SARS-CoV-2 Virus Neutralization Test

The antibodies’ neutralizing capacity was assessed using a commercial SARS-CoV-2 surrogate virus neutralization test (sVNT) (GenScript cPass™ SARS-CoV-2 Neutralization Antibody Detection Kit, Genscript, Piscataway, NJ, USA). This functional assay is based on the neutralizing antibody capacity to bind the receptor binding domain (RBD) of the spike protein and prevent its interaction with ACE2 receptors. Two different SARS-CoV-2 Spike protein RBD-HRPs were used: one mimicking the wild type virus RBD, and another for Omicron variant RBD. The method was performed as previously described by Meyer B. et al. [14]. Briefly, plasma samples, positive and negative controls, were diluted 1:10, mixed with equal volumes of enzyme-conjugated RBD, and incubated at 37 °C for 30 min. Subsequently, these mixtures were transferred to ACE-2-coated plates and incubated for 15 min. After plate washing, HRP-RBD–antibody complexes were removed, and the enzyme substrate (100 µL tetramethylbenzidine) was added for 15 min at room temperature. The optical density (OD) was assessed at 450 nm. Each sample’s neutralizing capacity was calculated using the following formula: % inhibition = (1 − (OD450 of sample/Average OD450 of negative controls) ×100. A value >30% inhibition was considered positive for neutralizing activity.

### 2.4. Data Processing and Statistical Analysis

Data were analyzed using GraphPad Prism version 9 software (GraphPad Software, La Jolla, CA, USA). Subjects were grouped according to age (<35, 35–50, 51–65, and >65 years), previous COVID-19 disease form (asymptomatic, mild, moderate, and severe), body mass index (BMI) (underweight (<18.5), normal (18.5–24.9), overweight (25–29.9), and obese (>30), sex (male and female), and the presence of comorbidities (such as diabetes, autoimmune disease, malignancies, and hypertension). The kinetics of the antibodies’ response were analyzed in all groups. In subjects with prior infection, anti-NCP IgG analyses were performed, taking into account the time since diagnosis confirmation. Antibody levels were expressed as medians with a 95% CI. Statistical differences between sample collection dates were assessed using a one-way Analysis of Variance (ANOVA), followed by a Tukey multiple comparisons test. For correlations between two or multiple groups, the Pearson r correlation coefficient was used. Statistical differences between characteristics of the two study groups were assessed using an Unpaired *t* test. All tests were performed with a nominal significance threshold of *p* < 0.05.

## 3. Results

### 3.1. Characteristics of the Study Population

A total of 571 participants were followed for 12 months after the first blood collection, which took place 1 month after the second vaccine dose. A total of 403 (70.58%) study participants received a third dose of vaccine 8 months after completion of the primary scheme (1 month before the 9-month follow-up).

The median age of study subjects was 46 years (95% CI: 37–52); only 2.45% were older than 65 years, and 13.84% had at least one comorbidity, most frequently an autoimmune disease.

Of all study subjects, 155 (27.15%) were positive for anti-NCP IgG antibodies (Table 1), out of which 90 individuals were previously diagnosed with SARS-CoV-2 infection by reverse transcription-polymerase chain reaction (RT-PCR). Samples from these 90 subjects were used to analyze anti-NCP antibodies’ dynamics.

There were no other significant differences between the baseline characteristics of participants with and without a prior SARS-CoV-2 infection, other than a lower BMI index in those previously naive (*t* test, *p* = 0.0384).

Of all study subjects, 155 (27.15%) were positive for anti-NCP IgG antibodies, (Table 1), out of which 90 individuals had been previously diagnosed with SARS-CoV-2 infection by reverse transcription-polymerase chain reaction (RT-PCR). Samples from these subjects were used for the analysis of anti-NCP antibodies’ dynamics.

### 3.2. Kinetics of Anti-SARS-CoV-2 Antibodies (Anti-Spike IgG, Anti-Spike IgA, Anti-NCP IgG)

**Figure 1:**
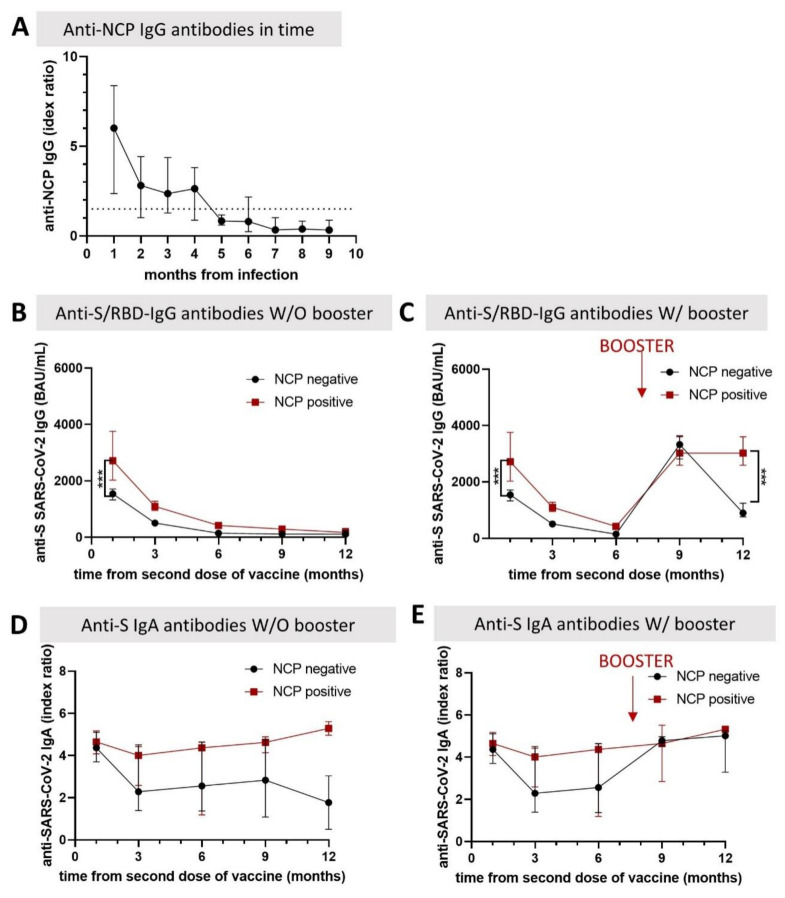
Dynamics of anti-SARS antibodies in vaccinated healthcare workers, Romania, 2021. Before the 9-month collection, 70.58% of study participants received a third dose of BNT162b2 mRNA vaccine (Pfizer-BioNTech). (**A**) Anti-NCP-IgG dynamic monitored from infection time, demonstrating a complete loss of antibodies 5 months post-infection; (**B**) The 12-month anti-S/RBD IgG antibody dynamic in individuals without a booster dose of BNT162b2 mRNA vaccine, showing an important decrease one year after vaccination, irrespective of the presence of a previous infection; (**C**) The 12-month anti-S/RBD IgG antibody dynamic in individuals who received three doses of BNT162b2 mRNA vaccine (at day 0, 3 weeks, and 8 months), showing the transitory effect of a booster dose after 3 months in naive individuals and a more persistent effect in those previously infected; (**D**) The 12-month IgA anti-spike dynamic in individuals without a booster dose of BNT162b2 mRNA, showing persistence only in those with a prior SARS-CoV-2 infection; (**E**) The 12-month IgA anti-spike dynamic in individuals with three doses of BNT162b2 mRNA vaccine, showing constantly increased levels in all participants after the booster dose. W/O, without; W, with. *** indicates *p* < 0.001.

The kinetics of different types of anti-SARS-CoV-2 antibodies are illustrated in Figure 1. In previously infected subjects, the anti-NCP IgG levels were correlated with the time span after infection, reaching undetectable levels 5 months after infection (Figure 1A).

The median anti-S/RBD IgG antibody level decreases significantly in all participants between 1 and 6 months post-vaccination (*p* < 0.001) and reaches a plateau thereafter. Nevertheless, people who were previously infected with SARS-CoV-2 initially developed, and maintained over time, significantly higher anti-S/RBD antibody levels after vaccination, compared to those who were SARS-CoV-2 naive at 6 months after the second dose of vaccine. SARS-CoV-2 naive subjects displayed only 9.6% of the initial IgG level, while those with a prior infection retained 15.5% of the post-vaccination level (Figure 1B). The booster dose restores antibody levels to the initial values yielded by the second dose at the same time interval in all subjects. Nevertheless, in naive individuals, the follow-up dynamic after the booster dose is similar to that displayed after the second dose, with a significant decrease at 3 months, while subjects with a prior infection maintain high anti-S IgG levels (Figure 1C).

The anti-S IgA antibody level, a surrogate indicator of mucosal immunity, decreases in previously naive individuals 3 months post-vaccination, reaching 40.7% of the initial level, with a further drop-off at 12 months in those who did not receive a booster dose. On the contrary, vaccination after natural infection induces a sustained, long-lasting IgA response, even without a booster dose of vaccine (Figure 1D).

### 3.3. Breakthrough Infections Data

**Figure 2:**
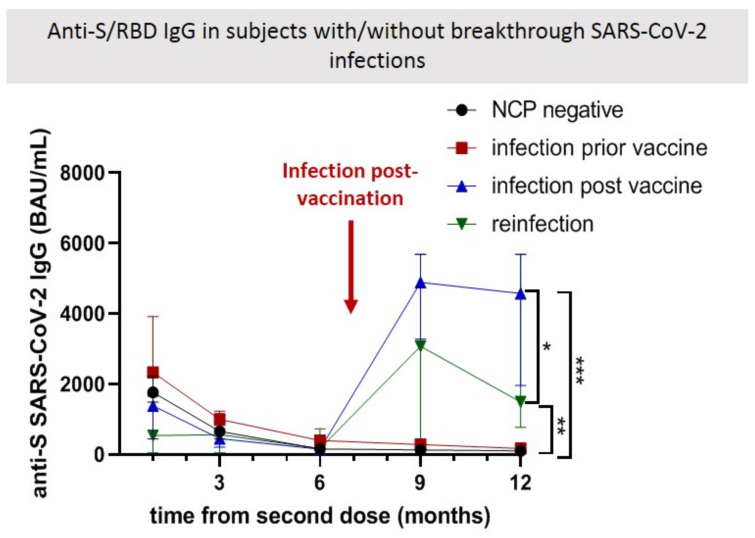
Anti-S/RBD IgG antibodies levels in vaccinated healthcare workers with breakthrough SARS-CoV-2 infections. Comparative antibody levels were depicted for 12 months, showing similar IgG anti-spike levels in individuals with and without a breakthrough infection at the 6-month collection point, but significant lower initial antibody titers in those infected prior to vaccination who experience breakthrough infections (blue and green lines) compared to those who do not (black and red lines). A red arrow indicates the moment of infection in vaccinated individuals (all infections acquired during the Delta VOC wave in Romania). At 12 months post-vaccination, significantly higher antibody levels are present in all patients who experience breakthrough infections compared with those who remain uninfected (*p* < 0.001), with superior and more stable titers maintained in those uninfected prior to vaccination (*p* < 0.05). * indicates *p* < 0.05; ** indicates *p* < 0.01; *** indicates *p* < 0.001.

Breakthrough infections were diagnosed starting at 6 months post-vaccination in 38 (6.65%) participants, of whom 22 (3.85%) were previously naive, and 16 (2.80%) were infected with SARS-CoV-2 prior to vaccination. In order to understand if the anti-S/RBD IgG antibody level can serve as a correlate of protection, we compared the prior anti-S/RBD IgG antibody levels between HCW with and without (w/wo) a breakthrough infection at equivalent time points (Figure 2). At the time of infection, the mean anti-S/RBD IgG antibody level in HCW who experienced breakthrough infections is similar to that registered in those who remained uninfected (156.4 BAU/mL [95% CI: 123.2–285.5] vs. 166.3 BAU/mL [95% CI: 119.1–242.6]) (*p* = 0.999). Interestingly, HCW who were infected prior to vaccination, and reinfected at 6 months post-vaccination (16 persons) develop lower initial levels of anti-S IgG antibody (at 1 month post-vaccination) than other subjects (543.46 BAU/mL vs. 23333.50 BAU/mL in those with prior infection, and 1768.26 BAU/mL in those with no infection prior to vaccination); however, this difference disappears at 6 months post-vaccination. The reinfected individuals develop and maintain lower antibody titers after the breakthrough infection compared to those infected for the first time after vaccination (*p* < 0.05). This may suggest that individual differences in primary immune responses can play an important role in long-term protection.

### 3.4. Factors That Influence Antibody Dynamics

The influence of several host factors on the dynamics of the humoral response was analyzed.

**Figure 3:**
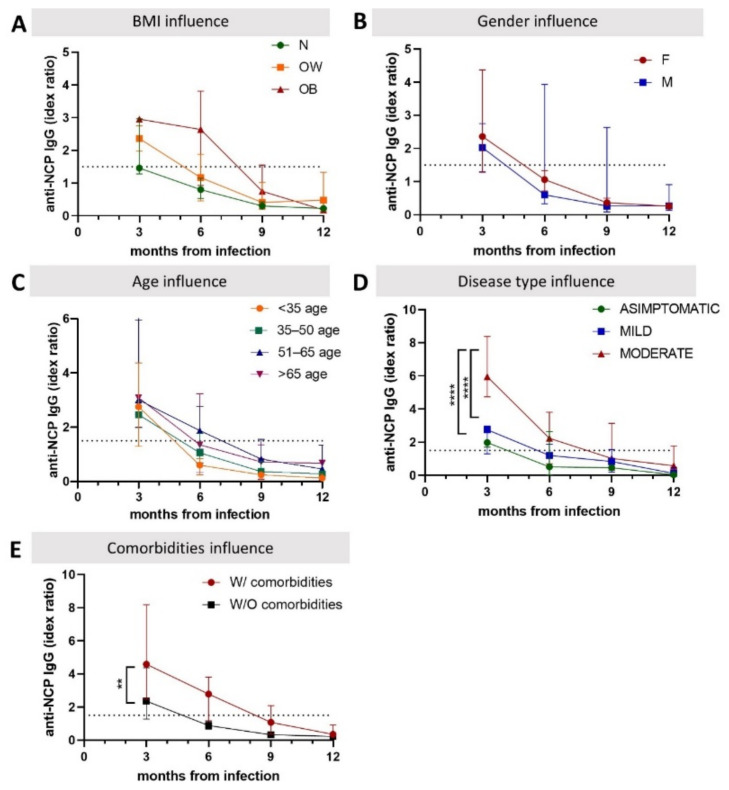
Host factors’ influence on anti-NCP IgG antibodies in healthcare workers, Romania, 2021. Influence of BMI (**A**), gender (**B**), age (**C**), disease form (**D**), and presence of comorbidities (**E**) was analyzed. N, normal weight; OW, overweight; OB, obese; W/O, without; W, with; F, female; M, male. ** indicates *p* < 0.01; **** indicates *p* ≤ 0.0001.

Higher index values for anti-NCP IgG antibodies are found in people with a higher BMI index, who have a mild form of the disease, and in those with comorbidities (Figure 3). In all of them, the antibodies tend to be maintained for longer periods over time (6–7 months), with a slow decrease. No differences are observed in the index values for anti-NCP IgG levels according to gender or age.

**Figure 4:**
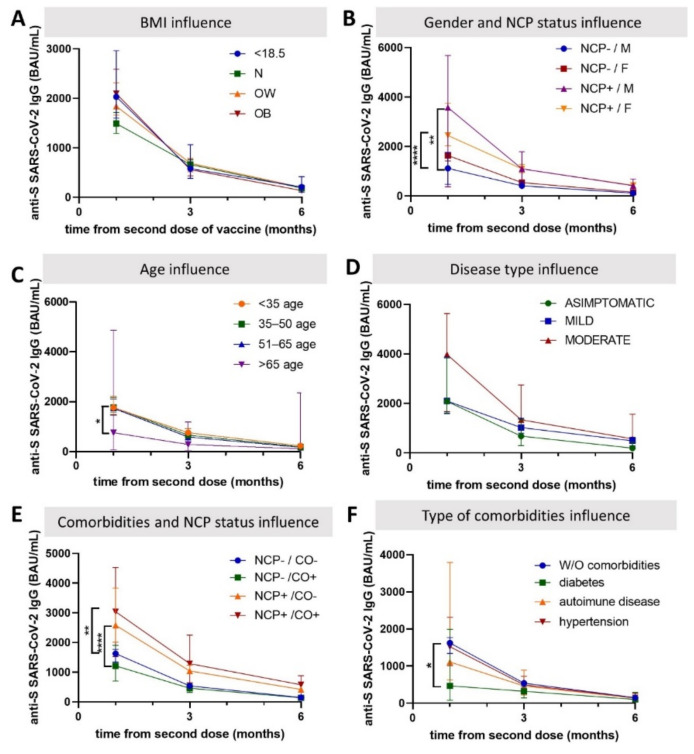
Host factors’ influence on the dynamics of anti-S/RBD IgG antibodies in healthcare workers, Romania, 2021. The influence of cofactors such as BMI (**A**), gender (**B**), age (**C**), COVID-19 disease form (**D**), presence of comorbidities (**E**), and type comorbidity (**F**) was analyzed. NCP negative (-), SARS-CoV-2 naïve; NCP positive (+), with prior SARS-CoV-2 infection; N, normal weight; OW, overweight; OB, obese; W/O, without; W, with; F, female; and M, male. * indicates *p* < 0.05; ** indicates *p* < 0.01; **** indicates *p* ≤ 0.0001.

Slightly elevated values of anti-S/RBD IgG antibodies are observed in previously infected HCW who experienced a moderate form of infection compared to those with asymptomatic or mild infections, with a slower, but still significant, decline at 6 months (Figure 4). Significantly lower antibody levels are observed in subjects over 65 years of age at 1 month post-vaccination compared to the other age groups. Although the proportion of older subjects is limited, no statistically significant differences are maintained at 3 months post-vaccination. Similar antibody levels are present irrespective of the subject’s BMI and presence of comorbidities, with the exception of subjects with diabetes who have significantly lower levels at 1 month post-vaccination. There are no differences in the anti-S IgG antibodies according to gender in previously uninfected HCW; in those vaccinated after a previous infection, levels are 1.69-fold higher in males compared to females.

### 3.5. The Neutralizing Capacity

Neutralizing capacity against the ancestral variant is maintained over time in subjects who received a booster dose (median 93.58% [95% CI: 86.98–94.06]), while it decreases slowly, but significantly, at 12 months after the second dose in subjects without a booster dose (median 88.92% [95% CI: 85.48–90.97]). Neutralizing capacity against the Omicron variant is significantly lower compared to the ancestral variant (77.07% [95% CI: 25–82.65]) in subjects without booster doses, but still significant in HCW with booster doses (82.75% [95% CI: 76.17–87.04%]). There is no difference in terms of neutralizing capacity for individuals with breakthrough infections compared with those who remained uninfected (*p* = 0.610).

**Figure 5:**
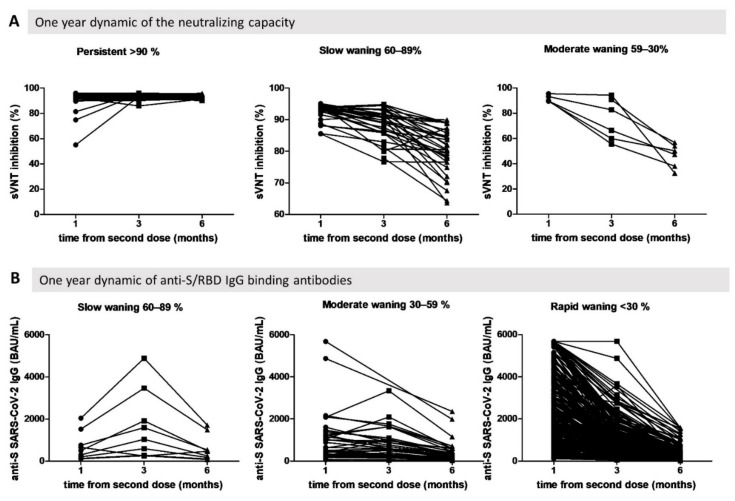
Modeling waning immunity for HCW, Romania, 2021. Four distinctive dynamics patterns reported at the initial level: persistent (>90%), slow (60–89%), moderate (30–59%), and rapid waning (<30%). (**A**) Neutralizing antibody dynamics, measured by percentage of sVNT readings of sVNT. (**B**) Anti-S/RBD IgG levels measured in BAU/mL.

Using temporal changes in neutralizing antibodies levels and interpretation thresholds indicated by the manufacturer of the sVNT kit, three distinctive patterns of neutralizing antibody dynamics were identified: persistent immunity (>90%), slow decrease (60–89%), and moderate decrease (30–59%). None of the participants had a rapid decrease in neutralizing capacity (below 30%); instead, for the majority (68.2%), the neutralization capacity against the ancestral variant was preserved over time, 27.6% had a slow decrease, and only 4.13% had a moderate decrease in neutralizing capacity (Figure 5A).

At the same time, the vast majority of participants (87.39%) experienced a rapid decrease in anti-S/RBD IgG levels, 10.03% experienced a moderate decrease, and only 2.58% had a slow waning pattern. Interestingly, participants who tend to maintain anti-S/RBD IgG overtime had lower initial levels of antibody 1 month after the initial vaccination regimen (Figure 5B).

## 4. Discussion

In this study we report the dynamics of the anti-SARS-CoV-2 antibody response during a one year period following vaccination with the BNT162b2 vaccine, (two doses administered 3 weeks apart, with or without a booster dose at 8 months) in HCW from Romania.

We confirm previous reports [10] of a marked and continuous decline of anti-S/RBD IgG antibodies during the first 6 months post-vaccination, followed by a plateau until 12 months, and the short-term boosting effect of a booster shot [14].

At 3 months post-booster dose we report a marked decline in the anti-S/RBD IgG levels in individuals without SARS-COV-2 infection prior to vaccination, compared to those previously infected. These results are in agreement with other studies that suggest a limited time effect of the booster dose on the antibody level [15]. However, after the booster shot the neutralizing capacity against the ancestral variant remains high in the majority of patients and significant against the immune evasive Omicron variant, supporting the role of a booster dose against severe disease. In addition, the anti-Spike IgA antibodies, a surrogate marker of mucosal immunity, remain at high levels in boosted individuals, irrespective of their previous exposure to infection. This may confer further protection in persons receiving three doses of an mRNA vaccine, as lower serum levels of anti-Spike IgA were correlated with prevention of breakthrough infections with SARS-CoV-2 variants [16]. However, inducing superior anti-SARS-CoV-2 mucosal immunity using innovative vaccines remains an important goal in stopping viral transmission. Several preclinical studies show that intranasal or orally administered adenoviral-based vaccines can protect against transmission and reduce disease severity [17], inducing mucosal specific IgA and tissue-resident memory cells [18].

A plethora of studies demonstrate that both natural infection and vaccination induce strong, yet short lived, protection against reinfection. Some studies suggest that immunity after infection may offer even better protection against reinfections compared to vaccination with two doses of an mRNA vaccine, but data are not fully consistent [9]. In a national survey in Denmark, there was no evidence that protection against reinfections was waning after 6 months, a result consistent with data from Austria [19,20]. Interestingly, some studies show that protection against reinfection decreases 4–5 months after the initial infection, but increases thereafter. These observations can be explained by the possibility of a persistent viral load, and thereafter misreporting a prolonged SARS-CoV-2 infection as a reinfection [21,22], or by the time required for antibody affinity maturation. Reports regarding differences in antibody kinetics between persons recovered from SARS-CoV-2 infection and those who are vaccinated suggest that neutralizing antibodies and RBD-specific memory B cells persist for longer periods, and decrease modestly 8–10 months after infection in recovered persons [7]; however, other studies suggest that vaccination induces more potent cellular immune responses compared to natural infection after one year [23]. Nevertheless, for now, hybrid immunity, elicited by the combination of a prior SARS-CoV-2 infection followed by vaccination, seems to confer the greatest protection against symptomatic and severe SARS-CoV-2 infections [24]. Differences in the magnitude, persistence, and extent of humoral immune responses were observed in patients vaccinated after a prior infection compared to naive vaccine recipients [25].

A study by Levin et al. about waning immunity after vaccination concluded that antibody levels substantially decrease 6 months after vaccination with two doses, especially in elderly men and immunocompromised persons; unexpectedly, it is maintained at high levels in obese persons [7].

Little is known about the risk factors that identify people prone to infections. Some reports indicate high rates of reinfection in the elderly, immunocompromised patients, those with severe comorbidities, or subjects at risk of exposure (e.g., healthcare workers) [19,26,27]. In our study, only a small percentage of HCW developed symptomatic SARS-CoV-2 infections during the first 12 months after vaccination. These results can be explained by the fact that HCW are disproportionately exposed to high viral loads in hospitals, despite a constant use of personal protective equipment; this continuous contact with a natural antigenic stimulus can amplify the protection induced by vaccination. Studies of the general population suggest that neutralizing antibody levels were highly correlated with immune protection from symptomatic SARS-CoV-2, although no definitive cut-off was established [28]. In our study, there was no significant difference in the anti-Spike IgG level, nor in the neutralizing capacity between those with and without breakthrough infections. However, the number of breakthrough infections is limited; thus, most subtle individual differences in the innate or acquired immune responses can be considered.

It is important to mention that our study was conducted during a period of high prevalence of the Delta variant, and only shortly after the emergence of the Omicron variant. In the past few months, real-world studies illustrate that vaccine efficacy against Omicron symptomatic infections is close to non-existent/very low, while protection against hospitalization for severe disease is maintained, although at lower levels compared to that against the previously dominant Delta variant [29,30,31]. Long-term protection will depend on the emergence of viral variants with a high capacity for immune evasion, clustering in different serotypes from the previously circulating ones. Recent studies show that all VOCs, except the Omicron variant, cluster in a common serotype, explaining the efficacy of the original vaccine formulation against the Alpha and Delta variants [32].

The continuous antigenic drift of the Omicron VOC, with periodic displacement of circulating sub-lineages by others that are more immune evasive and as such, more prone to spreading in a highly immunized population, will raise important public health problems, with periodic waves of infections and potential severe consequences in highly vulnerable populations. Recent studies suggest that in the presence of BA.2, BA.4, and BA.5 Omicron subvariants, protection against severe disease is decreased in recipients of two vaccine doses and in previously infected, but unvaccinated persons.

Moreover, unvaccinated individuals who acquire an infection with one of the initial sub-lineages of the Omicron variant (BA.1) develop low levels of cross-neutralizing antibodies and may be susceptible to reinfections with another sub-variant (BA.2, BA.4, BA.5) [31]. Antigenic cartography of current, emerging, and future variants will allow a better understanding of the level of cross-reactivity elicited by vaccination or previous exposure to different viral variants [33]; this will point to the most probable amino acid profile related to immune evasion, and possibly lead to better adapted vaccine formulations or more individualized approaches towards a heterologous vaccination scheme.

This study has a series of limitations. Study results may not be representative of the general population, as participants were mostly healthy, without age- or disease-related immunosuppression, and were more frequently exposed to natural infections in healthcare facilities. There is a certain age bias, as more than 47% of individuals enrolled in the study were aged 35–50 years, while the most vulnerable age group, persons >65 years, who might have a less robust immune response due to immunosenescence, are under-represented (2.4% of the study subjects). Yet, the enrolled population is representative of the age structure of the healthcare work force in Romania, and the dynamics of the immune response are relevant for further specific recommendations in the national vaccination strategy. The study stopped during the initial phase of the Omicron wave in Romania, in which most infections were registered in children and young adults; it is probable that many more reinfections with the highly transmissible and antigenically unrelated Omicron variant will also be recorded during the next few months in healthcare workers. It was not possible to monitor the dynamics of the humoral immune response at all points for all enrolled participants, as there were several missing samples for at least one of the collection points; nevertheless, data were available for a sufficient number of participants to ensure statistical significance as defined by the study methodology.

## 5. Conclusions

In conclusion, the present study shows that one year after vaccination there is a consistent and constant decrease in the level of anti-S/RBD antibody, even in persons who receive a booster dose 8 months after completion of the initial regimen. Nevertheless, people with a prior SARS-CoV-2 infection develop significantly higher levels of anti-S IgG antibodies and long lasting IgA anti-S antibodies after a booster dose compared to naive subjects. A significant neutralizing capacity against the ancestral and Omicron variant is maintained at least 3 months after a booster dose, and the number of breakthrough infections in HCW is limited, possibly reflecting a continuous antigenic stimulation following frequent exposures in their professional setting.

Establishment of an antibody level cut-off value as a correlate for protection remains elusive. There are no statistically significant differences between the anti-S/RBD IgG levels in subjects with and without breakthrough infections, suggesting that frequent boosting might not be the most appropriate action in immunocompetent persons. Differences in cellular or innate immunity, or even individual genetic differences, are probably more important to the evolution of an infection post-vaccination. Longitudinal studies of memory B cells’ response will inform future vaccination strategies, especially in the presence of a highly immune-evasive viral variant with a constant evolution via antigenic drift.

## Figures and Tables

**Table 1:** Characteristics of the study cohort, Bucharest, Romania, 2021 (*N* = 571).

	Total	Anti-NCP Negative	Anti-NCP Positive	*p* Value
	(*N* = 571)	(*N* = 416; 72.85%)	(*N* = 155; 27.15)	
**Sex**				
Female	485 (84.94%)	351 (84.37%)	134 (86.45%)	
Male	86 (15.06%)	65 (15.63%)	21 (13.55)	
**Age (years)**				0.5714
Median (95% CI)	46.00 (37–52)	45.50 (36–52)	46.00 (39–52)	
<35	117 (20.49%)	93 (22.36%)	24 (15.48%)	
35–50	273 (47.81%)	190 (45.46%)	83 (53.55%)	
51–65	167 (29.25%)	122 (29.33%)	44 (28.39%)	
>65	14 (2.45%)	11 (2.64%)	4 (2.58%)	
**BMI** ^a^				0.0384 *
Median (95% CI)	25.46 (21.99–29.64)	25.00 (21.82–29.39)	26.59 (22.31–29.90)	
Underweight (<18.5)	23 (4.20%)	20 (5.03%)	3 (2.00%)	
Normal (18.5–24.9)	235 (42.88%)	178 (44.72%)	57 (38.00%)	
Overweight (25–29.9)	165 (30.11%)	112 (28.14%)	53 (35.33%)	
Obese (>35)	125 (22.81%)	88 (22.11%)	37 (24.67%)	
**Comorbidities**				
Total	79 (13.84%)	56 (13.46%)	23 (14.84%)	
Diabetes	18 (22.78%)	13 (23.21%)	5 (21.74%)	
Malignancies	6 (7.59%)	4 (7.14%)	2 (8.70%)	
Autoimmune disease	24 (30.38%)	18 (32.14%)	6 (26.09%)	
Hypertension	17 (21.52%)	11 (19.64%)	6 (26.09%)	
Other	14 (17.72%)	10 (17.86%)	4 (17.39%)	
**Breakthrough SARS-CoV-2 infections**	38 (6.65%)	22 (5.29%)	16 (10.32%)	
		3.85%	2.80%	
**Booster dose**	403 (70.58%)	304 (73.08%)	-63.87%	

^a^ Missing data (total *N* = 548; NCP negative *N* = 398; NCP positive *N* = 150). * Unpaired *t* test.

## Data Availability

Not applicable.
